# Atrial fibrillation, sinoatrial and atrioventricular node dysfunction in a mouse model of heart failure with preserved ejection fraction

**DOI:** 10.1113/EP092628

**Published:** 2025-09-17

**Authors:** Bernadin Ndongson‐Dongmo, Reinhard Bauer, Finn Olav Levy

**Affiliations:** ^1^ Department of Pharmacology, Institute of Clinical Medicine University of Oslo Oslo Norway; ^2^ Department of Pharmacology, Division of Laboratory Medicine Oslo University Hospital Oslo Norway; ^3^ Institute of Molecular Cell Biology Jena University Hospital Friedrich Schiller University Jena Germany

**Keywords:** atrial fibrillation, atrioventricular block, chronotropic incompetence, diastolic dysfunction, heart failure with preserved ejection fraction, sinoatrial node dysfunction, sinus pauses

## Abstract

Millions of people are affected by atrial fibrillation (AF) and heart failure with preserved ejection fraction (HFpEF), two disorders frequently found simultaneously. However, the interrelationship between these intertwined disorders is poorly understood, partly owing to the lack of preclinical models. We aimed to evaluate whether a recently developed mouse model of HFpEF could also be used as a model of AF and, potentially, to study the co‐occurrence and interrelationship between the two conditions. Mice were fed a dietary regimen of high‐fat diet and *N* ^ω^‐nitro‐l‐arginine methyl ester in the drinking water to induce HFpEF. Twenty‐four‐hour ECG recordings acquired by telemetry were analysed for autonomic imbalance. After 24 h ECG recording, mice received isoprenaline, and a further 1 h of recording was assessed for chronotropic incompetence, susceptibility to atrial arrhythmia and conduction impairment. Evaluation of diastolic function was achieved by transcarotid catheterization and histological analysis performed on the hearts. Resting heart rate was significantly increased after 3 weeks of the dietary regimen, with a trend observed as early as 1 week. Premature atrial contractions, sinus pauses and atrioventricular blocks occurred significantly after 3 weeks of the dietary regimen. Significant diastolic dysfunction, chronotropic incompetence and higher occurrence of AF after isoprenaline stimulation were observed in the HFpEF group at 6 weeks of the dietary regimen. Our study revealed that sinoatrial node and atrial dysfunction precede the simultaneous occurrence of AF, diastolic dysfunction and chronotropic incompetence. This mouse HFpEF model might be helpful for studying the interdependence between AF and HFpEF.

## Abstract (highlights)

- **What is the central question of this study?**
  Could a recently developed two‐hit mouse model of heart failure with preserved ejection fraction (HFpEF) be suitable to study the simultaneous occurrence of atrial fibrillation (AF) and HFpEF?
- **What is the main finding and its importance?**
  This study demonstrates that the two‐hit mouse model of HFpEF displays susceptibility to AF and recapitulates some features of clinical HFpEF also linked to AF. These similarities to clinical HFpEF suggest that this model might be helpful for studying the mechanisms of the interdependence between AF and HFpEF.

## Graphical abstract

Summary of the timeline of characterized events related to atrial fibrilation (AF) and diastolic dysfunction in a mouse heart failure with preserved ejection fraction (HFpEF) model. Three weeks after the initiation of the diet regimen with high fat diet (HFD) and *N* ^ω^‐nitro‐l‐arginine methyl ester (l‐NAME) in drinking water to induce HFpEF, significantly higher resting sympathetic activity was observed. At high pharmacological sympathetic stimulation with isoprenaline after 3 weeks of diet regimen, a significantly higher susceptibility to premature atrial contraction (PAC) and conduction abnormalities revealed by sinus pauses and atrioventricular blocks. While similar to control at 3 weeks of diet regimen, the diastolic dysfunction and the susceptibility to atrial fibrillation at high pharmacological sympathetic stimulation was significantly higher after 6 weeks of diet regimen. These findings show that the simultaneous occurence of diastolic dysfunction and susceptibility to atrial fibrillation after 6 weeks of diet regimen is preceded by PAC and conduction abnormalities. This study highlights that this mouse model recapitulates some features of clinical HFpEF also linked with AF and may be helpful for studying the interdependence between AF and HFpEF.

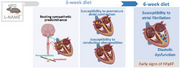

## 1 INTRODUCTION

Heart failure (HF) with preserved ejection fraction (HFpEF) is a clinical syndrome affecting about half of all HF patients (Pfeffer et al., 2019). HFpEF is characterized by the interaction of various cardiac and extracardiac conditions, resulting in a heterogeneous phenotypic spectrum (Gentile et al., 2022). HFpEF is therefore regarded as a systemic syndrome that goes beyond left ventricular (LV) diastolic dysfunction (Shah et al., 2020) and is subjected to intense research regarding the pathophysiological mechanisms underlying its generation and progression. Atrial fibrillation (AF), the most common sustained cardiac arrhythmia (Bapat et al., 2023), has been recognized to have an interdependence with HFpEF (Packer et al., 2020). HFpEF and AF frequently coexist and have interlinked clinical conditions (Kotecha et al., 2016; Sartipy et al., 2017). The convergence of HFpEF and AF contributes to poor outcomes, escalating morbidity and mortality. The advent of AF during HFpEF can worsen the clinical course by posing a higher risk of associated complications, including thromboembolic events and exacerbation of HF (Hoit, 2014; Kotecha et al., 2016). HFpEF and AF share risk factors and comorbidities such as ageing, hypertension, diabetes, obesity, sleep apnoea, pulmonary and renal diseases, all of which contribute to changes in cardiovascular characteristics, including myocardial and vascular stiffening (Kotecha et al., 2016; Paulus & Tschope, 2013; Sartipy et al., 2017). However, the pathophysiological mechanisms of the interrelationships between HFpEF and AF are underexplored, in part owing to lack of suitable preclinical models.

When coexisting, AF and HFpEF progression are so strikingly intertwined that the determination of cause and effect has not been easy to clarify. Three main hypothetical frameworks of explanation deriving from epidemiological and clinical studies have been advanced. The first one suggests that the increased LV filling pressure in HFpEF might cause left atrial (LA) functional, structural and electrical remodelling that can trigger AF, with LA fibrosis being the substrate linking these abnormalities. The second suggests that the arrhythmic component of AF might contribute to LV dysfunction. And the third suggests that the two disorders might be parallel manifestations of the same underlying myocardial disease, which causes AF (because it affects atria) and HFpEF (because it affects the LV) (Hoit, 2014; Kotecha et al., 2016; Naser et al., 2023). In addition, although AF and HFpEF are reported to be progressive conditions (Ariyaratnam et al., 2021; Heijman et al., 2021), little is known about the temporal initiation and the key events preceding AF during HFpEF development. As shown above, there has been significant progress from epidemiological and clinical reports to characterize the syndrome of AF and HFpEF. However, knowledge of paramount importance on pathophysiological mechanisms is missing and will require an animal model capable of recapitulating the clinical features of this syndrome. There are animal models with characterized diastolic dysfunction and some features of HFpEF (Doi et al., 2000; Hamdani et al., 2013; Schiattarella et al., 2019) or models with reported AF (Bapat et al., 2023; Kondo et al., 2018; Polina et al., 2020; Riley et al., 2012), but models reporting the simultaneous occurrence of the two disorders are lacking.

In the present study, we evaluate whether a mouse model of HFpEF (Schiattarella et al., 2019) driven by a dietary regimen consisting of high‐fat diet (HFD) plus drinking water containing *N* ^ω^‐nitro‐l‐arginine methyl ester (l‐NAME) recapitulates clinical features of interdependence between HFpEF and AF. We have characterized, in a time‐dependent manner, the occurrence of diastolic dysfunction and AF, in addition to several known pathophysiological risk factors and comorbidities of clinical HFpEF associated with these two disorders. Specifically, in addition to diastolic dysfunction and AF, we have monitored autonomic imbalance, premature atrial contractions, conduction impairment and chronotropic incompetence. We revealed a simultaneous occurrence of diastolic dysfunction, AF and chronotropic incompetence. We also revealed that these characteristics were preceded by premature atrial contractions and conduction system defects and, even earlier, by autonomic imbalance. Our study showed that the present mouse model is suitable for the study of pathophysiological mechanisms of the interdependence between HFpEF and AF, in addition to AF prediction and possible preventive intervention strategies.

## 2 MATERIALS AND METHODS

### 2.1 Ethical approval and animal welfare

Male C57BL/6N mice were used in this study. The animal procedures were performed according to the guidelines from Regulation (EU)2019/1010 of the European Parliament on the protection of animals used for scientific purposes and by the Norwegian Animal Welfare Act, conforming to this directive. The Norwegian Food Safety Authority approved the experiments (approval reference: 18/275205; FOTS ID 18024). Animals (from Janvier Labs) were kept in standard housing conditions with a 12 h–12 h light–dark cycle and free access to food and water.

### 2.2 Animal groups and dietary regimen

**FIGURE 1:**
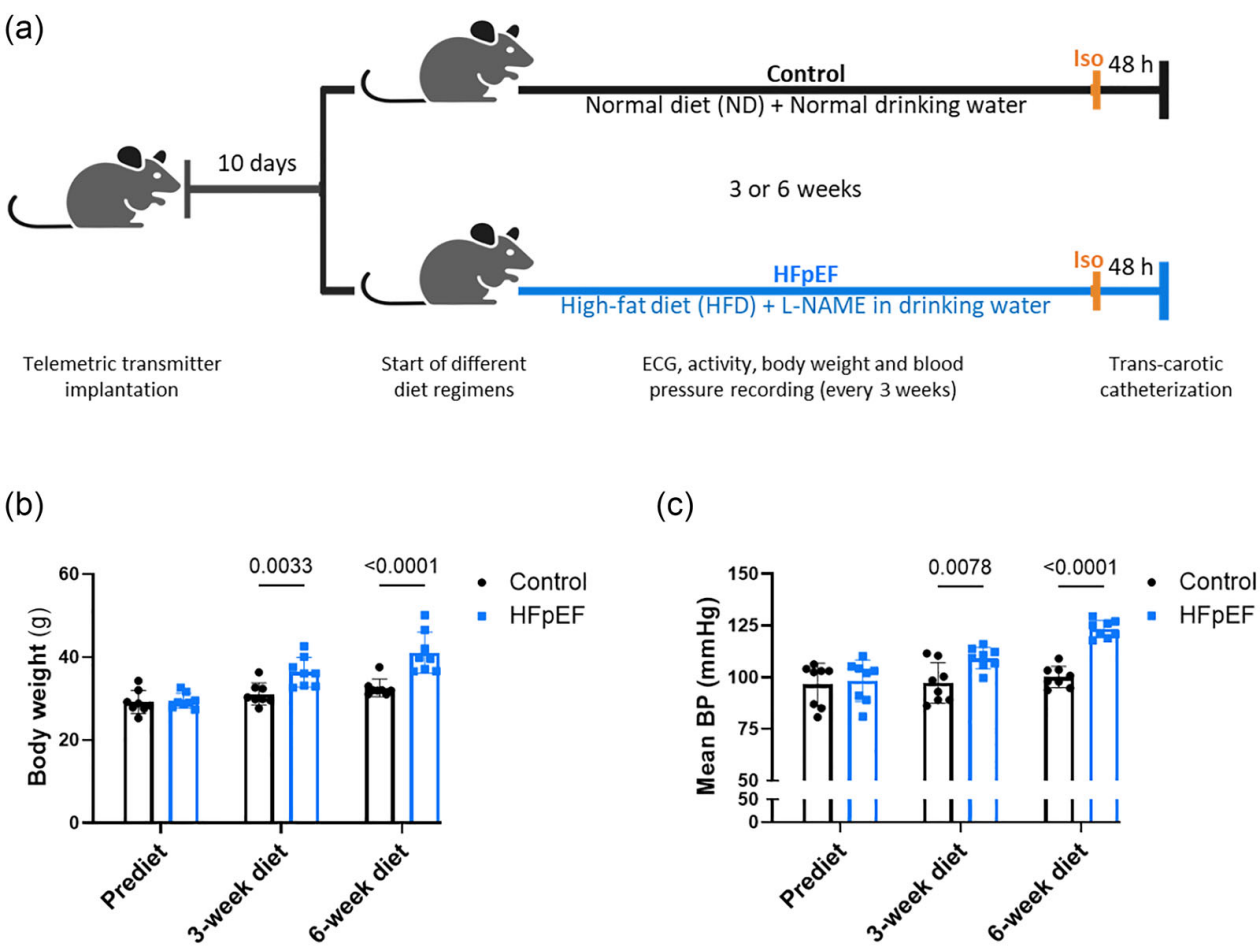
Experimental protocol and phenotypical verification of ‘two hits’. (a) Experimental protocol showing the time line of experimentation and data collection. Telemetry devices were implanted in 12‐week‐old C57BL/6N mice, and different dietary regimens started after full recovery (10 days). Two groups (a group for 3 weeks and a group for 6 weeks dietary regimen) of mice were monitored for body weight, blood pressure, 24 h ECG, locomotor activity and body temperature every 3 weeks. At the end of the dietary regimen period, all mice received an injection of isoprenaline (Iso), followed by 1 h ECG recording. Finally, trans‐carotic catheterization was performed ≥48 h after Iso injection. (b,c) The evolution of body weight (b) and blood pressure (c), measured every 3 weeks, in control and HFpEF groups from the day before (Prediet) to 6 weeks after initiation of the dietary regimen. Body weight and blood pressure were recorded in eight (*n* = 8) mice in each group. Values are the mean ± SD. Differences among groups were analysed by ordinary two‐way ANOVA followed by the Holm–Sidak *post hoc* test. Numbers above square brackets show *p*‐values.

Animals were assigned randomly to two groups after 10 days recovery from telemetry device implantation surgery, as illustrated in Figure 1a. The control group was maintained on standard diet (16% protein rodent diet, Teklad Global) and water, whereas the HFpEF group was put on a dietary regimen with HFD (rodent diet with 60% kcal fat, Research Diets) plus drinking water containing l‐NAME (Sigma Aldrich) at 0.5 g/L. The effect of the ‘two hits’ (HFD and l‐NAME) was verified by recording body weight and blood pressure before initiation of the different dietary regimens and after 3 and 6 weeks of the regimen.

### 2.3 Implantation of telemetric transmitter

Mice at 12 weeks of age were implanted with a telemetry transmitter [ETA‐F10, Data Sciences International (DSI)] intraperitoneally via an abdominal incision. Before incision, mice were anaesthetized by inhalation of 4% isoflurane and maintained during the procedure with 2.5% isoflurane (Zoetis Animal Health) in oxygen. Three minutes before incision, local anaesthesia was administered (bupivacaine, Baxter AS, 2 mg/kg s.c.) at the location of the incision and area of lead placement of electrodes. The two‐lead electrodes were positioned subcutaneously in a lead II configuration (negative electrode on the dorsal surface of the xiphoid process and positive electrode on anterior mediastinum close to the right atrium). This electrode location guarantees high‐quality ECG recordings, even during vigorous somatomotor activity. The opoid analgesic buprenorphine (Orion Pharma Animal Health) was administered to all animals (0.1 mg/kg s.c. 30 min before surgery, and every 8 h for 24 h after surgery). Body heat was maintained both during and immediately after surgery. Animals were given a standard diet and water postsurgery. Prior to the start of experimental recordings, mice were allowed to recover for 10 days.

### 2.4 Recording of telemetric signals

ECG, body temperature and locomotor activity signals were recorded continuously for 24 h before the start of the dietary regimen (prediet) with animals left undisturbed in their home cages. The same recording was repeated at 3 and 6 weeks after the initiation of the dietary regimen. Signals were picked up by platform receivers (RPC‐1, DSI) and transferred via DSI data exchange matrix (sampling rate of 2 kHz with 12‐bit precision) to the acquisition software (Ponemah Software v.6.51, DSI) and stored on a personal computer for off‐line data analysis.

### 2.5 Pharmacological sympathetic stimulation

At the end of the last 24 h recording (at 3 or 6 weeks of the dietary regimen), mice received (i.p.) pharmacological sympathetic stimulation with the β‐adrenergic agonist isoprenaline (Iso; Sigma Aldrich) using 0.1 mL of 1 mg/mL solution in normal saline, and recording was continued for an additional 90 min.

### 2.6 Analysis of heart rate and heart rate variability

Analysis was conducted using Ponemah software. The function or option ‘ECG Analysis Attributes’ was used to optimize the detection of QRS complexes, P and T waves and placement. The heart rate (HR) and RR intervals presented represent the average value of the preceding 30 min period. HR variability was analysed using variability analysis of time‐domain parameters, such as standard deviation of all normal RR intervals (SDNN) calculated for each 2 min segment of normal RR interval (normal sinus rhythm) of every hour and averaged for the resting or active period.

### 2.7 Analysis of arrhythmias

Susceptibility to arrhythmias was analysed in a 1 h ECG recorded after pharmacological sympathetic stimulation with Iso. Arrhythmias were reported as the number of events per hour. AF was defined as a rapid and irregular atrial rhythm (fibrillatory baseline in the ECG), with irregular RR intervals and absence of P waves on the surface ECG. AF was confirmed by recognizing premature atrial contraction (PAC) at the initiation. The number of AF episodes ≥5 s and the burden of AF characterized by cumulative time of AF during 1 h after Iso was reported. PAC was first selected using Data Insight, a specific tool of Ponemah software detecting several arrhythmic patterns. Sinus pauses, defined as RR interval more than 2‐fold of the preceding interval, and atrioventricular block (AV block), defined as escape of the QRS complex between two P waves, were also detected using Data Insight. Ventricular arrhythmic events were counted as the sum of ventricular ectopic beats and ventricular extrasystoles.

### 2.8 Analysis of chronotropic competence

Chronotropic competence was defined as the level of HR increase after pharmacological sympathetic stimulation with isoprenaline. Mean HR in the 30 min period after Iso injection was reported comparatively to mean HR in the 30 min before Iso injection. The responsiveness to Iso was calculated as the percentage of mean HR change from pre‐Iso to post‐Iso.

### 2.9 Measurement of blood pressure

Blood pressure was measured with the tail‐cuff method using a non‐invasive blood pressure system (Coda monitor, Kent Scientific) under light sedation (1.5%–2% isoflurane with 98.5%–98% O_2_) while maintaining the body temperature at 37°C. The system uses volume pressure‐recording sensor technology to measure the tail systolic, diastolic and mean blood pressure. Data were reported to a personal computer with a compatible data acquisition and management program (Kent Scientific's CODA Software, Kent Scientific).

### 2.10 Measurement and analysis of intraventricular pressure–volume relationship

Left ventricular catheterization was performed using a micro‐conductance pressure–volume catheter (1.2 Fr model FTH‐1212B‐4518, Transonic Europe BV) inserted via the right carotid artery. Before incision, mice were anaesthetized with 4% and maintained during the procedure with 2.5% isoflurane in oxygen. Three minutes before incision, local anaesthesia was administered (bupivacaine, Baxter AS, 2 mg/kg s.c.) at the location of incision. Carotid and aortic blood pressure were recorded during insertion, and basal LV pressure–volume traces were continuously registered in closed‐chest animals (Pacher et al., 2008). To obtain the cardiac intrinsic contractile and stiffness parameters, the application of vena cava occlusion was done after the basal recording. Acquisition and analysis of pressure–volume loops was performed using a PowerLab system (ADInstruments Ltd). This was a terminal procedure, and at the end of measurements, animals were euthanized by removal of the heart under deep general anaesthesia (3% isoflurane). Lung and heart weights were recorded and hearts kept either in 4% paraformaldehyde for histology or at −80°C.

### 2.11 Histology

Whole hearts for longitudinal section or the central segment (excluding the apex and base) for transverse section was fixed in a solution of 4% paraformaldehyde for paraffin histology (5‐µm‐thick sections). Longitudinal sections were stained with Haematoxylin and Eosin to measure the LA size, the septum and left ventricle lateral wall (LVLW), and to measure the cross‐sectional area of the cardiomyocytes in the left atrium (LA) and right atrium (RA). Transverse sections were stained with Haematoxylin and Eosin to quantify the cross‐sectional area of the cardiomyocytes in LV. Longitudinal sections were stained with TUNEL staining (Abcam) to evaluate the presence of fragmented DNA as a sign of apoptotic cells in the atrioventricular node (AVN), LA, RA and LV. Longitudinal sections were stained with Masson Trichrome staining (Polysciences) to evaluate the presence of myocardial fibrosis in the AVN, LA, RA and LV. Sections were scanned with an AxioScan Z1 (Carl Zeiss, Jena, Germany), in brightfield mode at a resolution of 0.11 µm per pixel. Zen Lite v.3.7 blue edition software (Carl Zeiss, Jena, Germany) was used for analysis. To measure LA and RA size, drawing around the LA and RA was used. To measure septum and LVLW thickness, the average of the width at the base, mid and apical region from Haematoxylin‐ and Eosin‐stained heart sections was used. To measure cell area, drawing around cross‐sections of cardiomyocytes was used in the atria (∼15) or ventricles (∼50), including the septum and posterior LVLW. To measure apoptotic cells, the TUNEL‐positive cells, characterized by dark brown and a black nucleus, were counted, and the ratio relative to the evaluated myocardial area was used. To measure fibrosis, the background was subtracted from measured blue colour intensity, and the ratio relative to the evaluated myocardial area was used. All measurements were performed in a blinded manner.

### 2.12 Statistical analysis

Numbers of animals and experiments, given in legends, are per group. Differences between two groups were analysed with Student's two‐tailed *t*‐test. For differences among three or more groups, ordinary one‐way or two‐way ANOVA followed by the Holm–Sidak *post hoc* test were used. All analyses were performed with GraphPad Software (Prism v.10.2.0). The *p*‐values from the tests are reported in the figures and were considered significant when *p* < 0.05.

## 3 RESULTS

### 3.1 Phenotypical verification of ‘two hits’ and cardiac diastolic dysfunction

**FIGURE 2:**
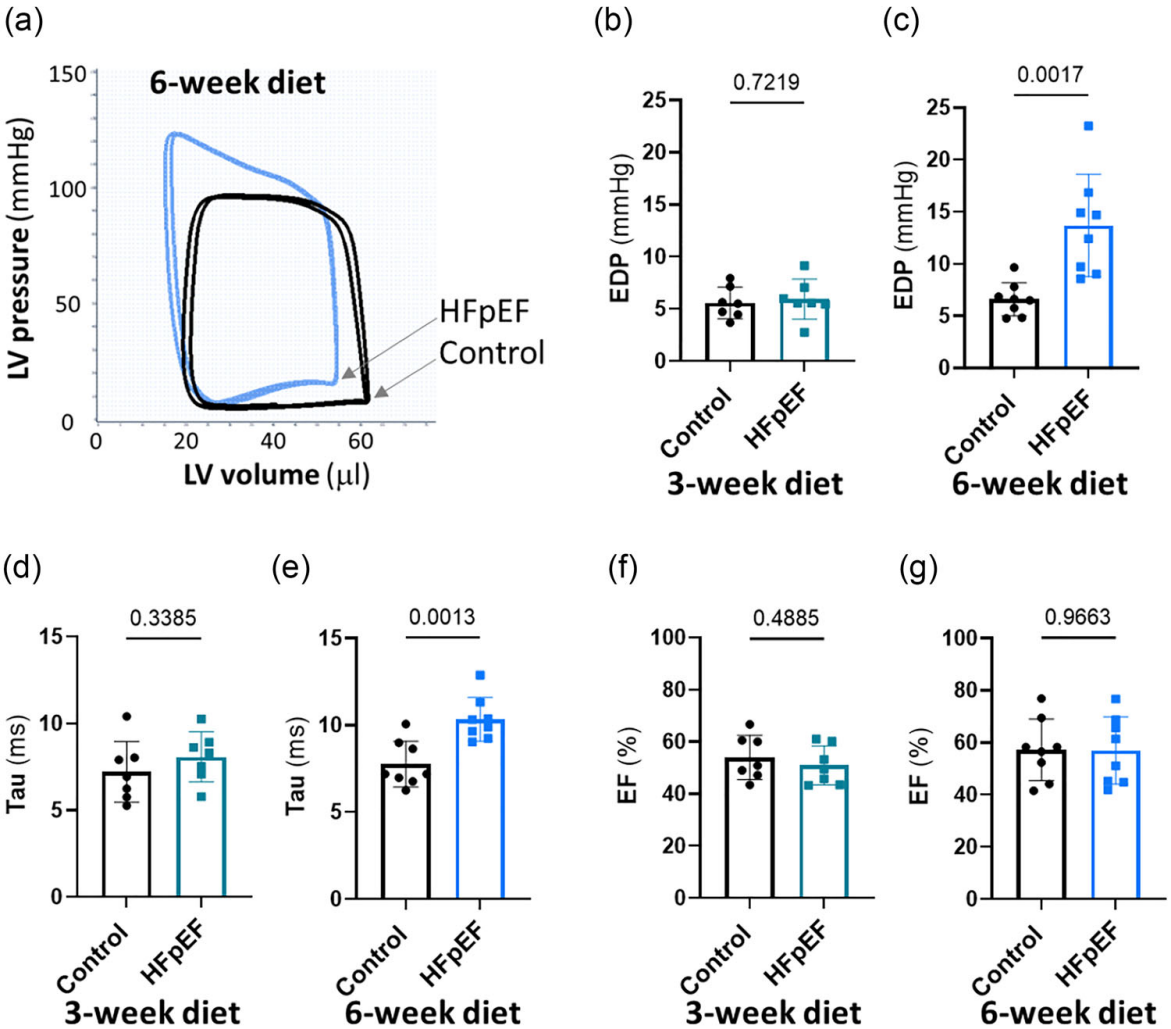
Diastolic dysfunction characterized by increased filling pressure and time of isovolumic relaxation in the early phase of heart failure with preserved ejection fraction (HFpEF). (a) Representative trace of left ventricular (LV) pressure–volume loops obtained from intracardiac catheter‐based measurement in control and HFpEF groups after 6 weeks of diet. (b,c) Mean data comparing filling pressure as end‐diastolic pressure (EDP) between control and HFpEF groups after 3 weeks (b) and 6 weeks (c) of the diet. (d,e) Mean data comparing the ventricular relaxation as the time of isovolumic relaxation (Tau) between control and HFpEF after 3 weeks (d) and 6 weeks (e) of the diet. (f,g) Mean data comparing the ejection fraction (EF) between control and HFpEF groups after 3 weeks (f) and 6 weeks (g) of the diet. Experiments were performed in seven (*n* = 7, 3‐week diet) and eight (*n* = 8, 6‐week diet) independent mice in each group. Values are the mean ± SD. Differences between groups were analysed with Student's two‐tailed *t‐*test. Numbers above bars show *p*‐values.

**FIGURE 3:**
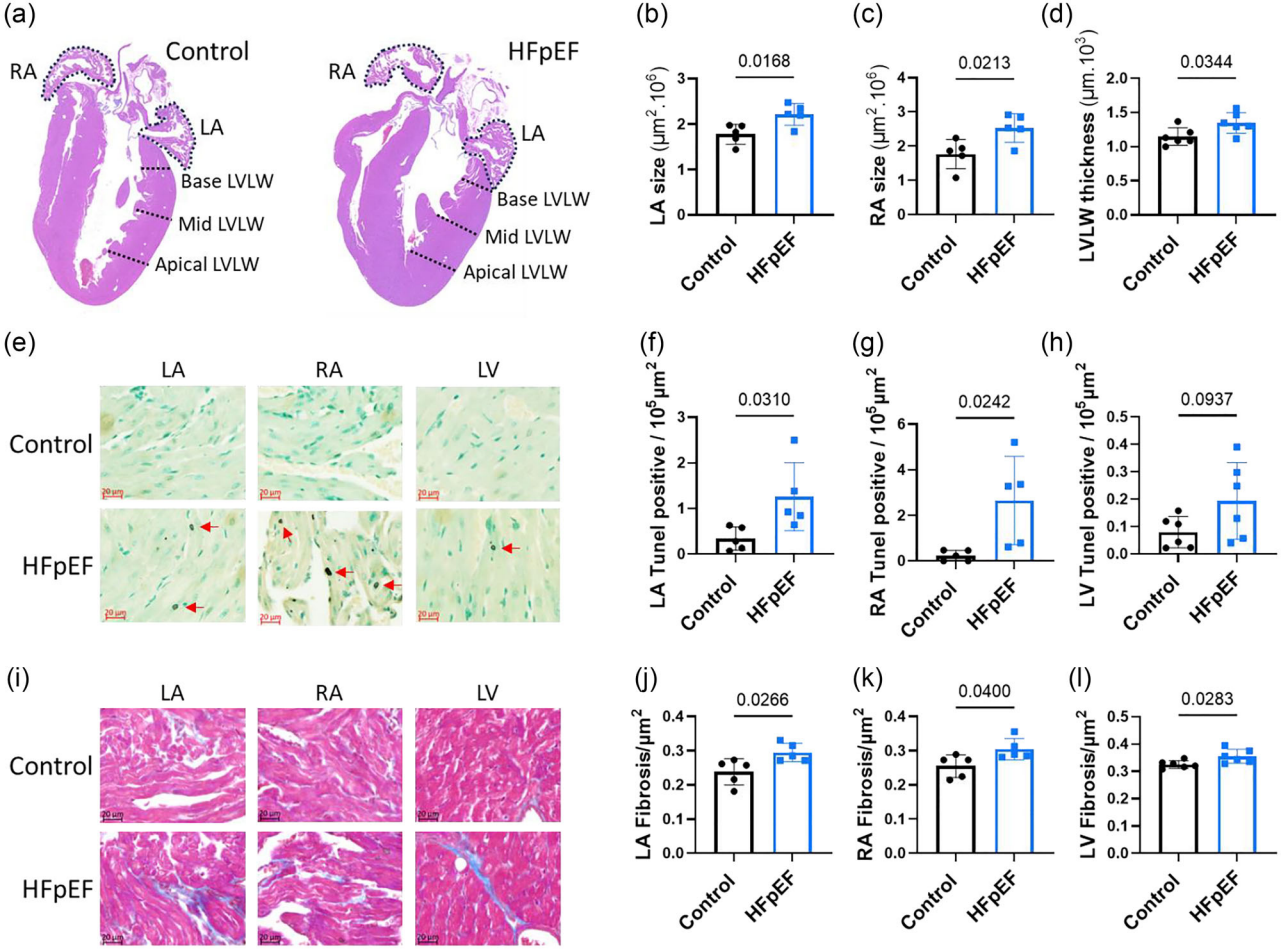
Morphological changes and/or myocardial injury and fibrosis in left atria (LA), right atria (RA) and left ventricles (LV). (a) Representative longitudinal heart sections stained with Haematoxylin and Eosin, showing measured LA and RA area (dotted delimitation), septum and LV lateral wall (LVLW) thickness (dotted lines) from control and heart failure with preserved ejection fraction (HFpEF) groups after 6 weeks of the diet. (b–d) Mean data comparing LA area (b), RA area (c) and LVLW (d). (e) Representative areas of TUNEL‐stained LA, RA and LV, showing apoptotic cells (red arrows), from control and HFpEF groups after 6 weeks of the diet. (f–h) Mean data comparing the number of apoptotic cells per tissue area between control and HFpEF in LA (f), RA (g) and LV (h). (i) Representative areas of Masson Trichrome‐stained LA, RA and LV, showing collagen level (blue colour intensity), from control and HFpEF groups after 6 weeks of the diet. (j–l) Mean data comparing the level of fibrosis (collagen) per tissue area between control and HFpEF groups in LA (j), RA (k) and LV (l). Evaluations were performed for each group independently in five (*n* = 5) LA, five (*n *= 5) RA and six (*n* = 6) LV. Values are the mean ± SD. Differences between groups were analysed with Student's two‐tailed *t‐*test. Numbers above bars show *p*‐values.

Initially, we sought to verify the effect of the dietary regimen; we recorded body weight for signs of obesity and blood pressure for signs of hypertension before and after 3 and 6 weeks (*n* = 8) of the dietary regimen (Figure 1a). The group of mice receiving HFD and l‐NAME in the drinking water (HFpEF group), progressively and significantly gained weight compared with the control group (Figure 1b), independently of tibial length (Table 1). In a similar manner to body weight, the blood pressure increased progressively, as shown in Figure 1c. The end‐diastolic pressure, which represents the LV filling pressure, was similar in both groups at 3 weeks (*n* = 7) of dietary regimen, but significantly elevated in the HFpEF group after 6 weeks (*n* = 8) (Figure 2a–c). The time of isovolumic relaxation (Tau) was also similar at 3 weeks of dietary regimen and significantly elevated after 6 weeks (Figure 2d,e), confirming the impairment of relaxation. Ventricular stiffness was also shown, with elevated end‐diastolic pressure–volume relationship compared with the control group after 6 weeks (Table 2), and the ejection fraction was preserved (Figure 2f,g). These data revealed diastolic dysfunction occurring after 6 weeks of the intervention and following earlier signs of obesity and hypertension. The weight of the ventricles, reflecting cardiac hypertrophy, and the lung weight, reflecting congestion, were significantly increased (Table 1). Increased LV lateral wall thickness (Figure 3d), LV cardiomyocyte size (Table 1) and LV fibrosis (Figure 3l) also confirmed ventricular hypertrophy and remodelling in the HFpEF group. LA and RA sizes were increased in the HFpEF group (Figure 3b,c), suggesting atrial remodelling. Liver and kidney weights were not increased (Table 1), indicating no further congestion, and the locomotor activity (Table 2) was similar between the two groups.

**TABLE 1:** Tibia length, weight of organs and additional histological evaluation.

Parameter	Diet for 3 weeks				Diet for 6 weeks			
	Control	HFpEF	*n*	*p*‐value	Control	HFpEF	*n*	*p*‐value
TL, mm	17.84 ± 0.5	17.74 ± 0.4	7	0.6618	17.76 ± 0.5	17.56 ± 0.5	8	0.4169
Lung weight/TL, mg/mm	7.44 ± 0.5	7.61 ± 0.3	7	0.4597	7.93 ± 0.4	8.55 ± 0.5	8	0.0222
Ventricles/TL, mg/mm	6.81 ± 0.4	6.97 ± 0.7	7	0.6441	7.14 ± 0.4	7.70 ± 0.4	8	0.0110
Liver weight/TL, mg/mm	61.73 ± 8.6	58.84 ± 7.5	7	0.5146	68.50 ± 12.9	56.20 ± 6.0	8	0.0290
Kidney weight/TL, mg/mm	18.92 ± 1.2	18.07 ± 1.4	7	0.2494	20.06 ± 2.1	18.78 ± 1.4	8	0.1821
Transverse section								
Septum thickness, mm	–	–	–	–	1466 ± 147	1550 ± 199	8	0.1794
LV CM area, mm^2^	–	–	–	–	370 ± 52	445 ± 70	8	0.0300
Longitudinal section								
Septum thickness, mm	–	–	–	–	995 ± 117	1082 ± 182	6	0.3847
LA CM area, mm^2^	–	–	–	–	117 ± 13.5	149 ± 29.2	5	0.0599
RA CM area, mm^2^	–	–	–	–	106 ± 15.3	112 ± 14.8	5	0.6167

*Note*: Differences between groups were analysed with Student's two‐tailed *t*‐test. Values are the mean ± SD. Abbreviations: CM, cardiomyocytes; HFpEF, heart failure with preserved ejection fraction; LA, left atrium; LV, left ventricle; RA, right atrium; TL, tibia length.

**TABLE 2:** Additional haemodynamic parameters, locomotor activity, heart rate variability parameters and premature ventricular contraction events.

Parameter		Diet for 3 weeks				Diet for 6 weeks			
		Control	HFpEF	*n*	*p*‐value	Control	HFpEF	*n*	*p*‐value
HR, beats/min		480 ± 16	474 ± 23	7	0.5771	429 ± 25	462 ± 17	8	0.0086
ESP, mmHg		84.2 ± 4.4	91.2 ± 6.4	7	0.0341	86.7 ± 8.4	106.5 ± 11.7	8	0.0017
SW, mmHg mL		2157 ± 242	2138 ± 336	7	0.9054	2740 ± 478	2717 ± 533	8	0.9282
CO, mL/min‐		12 865 ± 1600	11 753 ± 2400	7	0.3278	13 320 ± 1901	14 025 ± 2456	8	0.5312
d*P*/d*t*_max_, mmHg/s		7085 ± 1033	6950 ± 722	7	0.7822	6867 ± 1077	6816 ± 922	8	0.9208
d*P*/d*t*_min_, mmHg/s		−6782 ± 1358	−6095 ± 893	7	0.2856	−6433 ± 1395	−5785 ± 893	8	0.2873
EDPVR, mmHg/mL		0.32 ± 0.2	0.36 ± 0.2	5	0.7450	0.22 ± 0.1	0.60 ± 0.2	7	0.0016
ESPVR, mmHg/mL		4.23 ± 1.1	4.20 ± 0.6	5	0.9615	4.30 ± 0.7	4.40 ± 0.8	7	0.8128
MAP, mmHg		90.0 ± 6.0	99.5 ± 6.3	7	0.0124	85.3 ± 10.4	109.3 ± 9.2	8	0.0002
ATA2, counts/min	Resting	–	–	–	–	2.52 ± 0.9	2.99 ± 1.1	8	0.9304
	Active	–	–	–	–	5.98 ± 2.1	6.78 ± 1.9	8	0.7494
RR, ms	Resting	–	–	–	–	128.4 ± 6.3	118.2 ± 4.3	8	0.0029
	Active	–	–	–	–	114.7 ± 5.8	107.8 ± 3.3	8	0.0340
SDNN		–	–	–	–	14.09 ± 1.9	11.64 ± 1.5	8	0.0136
Vec, number/h		3.85 ± 2.9	5.28 ± 3.5	7	0.4261	6.50 ± 3.3	9.50 ± 8.1	8	0.3504

*Note*: Differences between groups were analysed with Student's two‐tailed *t*‐test. Values are the mean ± SD. Abbreviations: ATA2, locomotor activity; CO, cardiac output; d*P*/d*t*_max_, positive peak rate of pressure change (rise); d*P*/d*t*_min_, negative peak rate of pressure change (decline); EDPVR, end diastolic pressure‐volume relationship; ESP, end‐systolic pressure; ESPVR, end systolic pressure‐volume relationship; HFpEF, heart failure with preserved ejection fraction; HR, heart rate; MAP, mean arterial pressure; RR, RR intervals; SDNN, standard deviation of all normal RR intervals; SW, stroke work; Vec, ventricular ectopic beats and extrasystoles.

### 3.2 Susceptibility to atrial fibrillation and atrial contractile dysfunction

**FIGURE 4:**
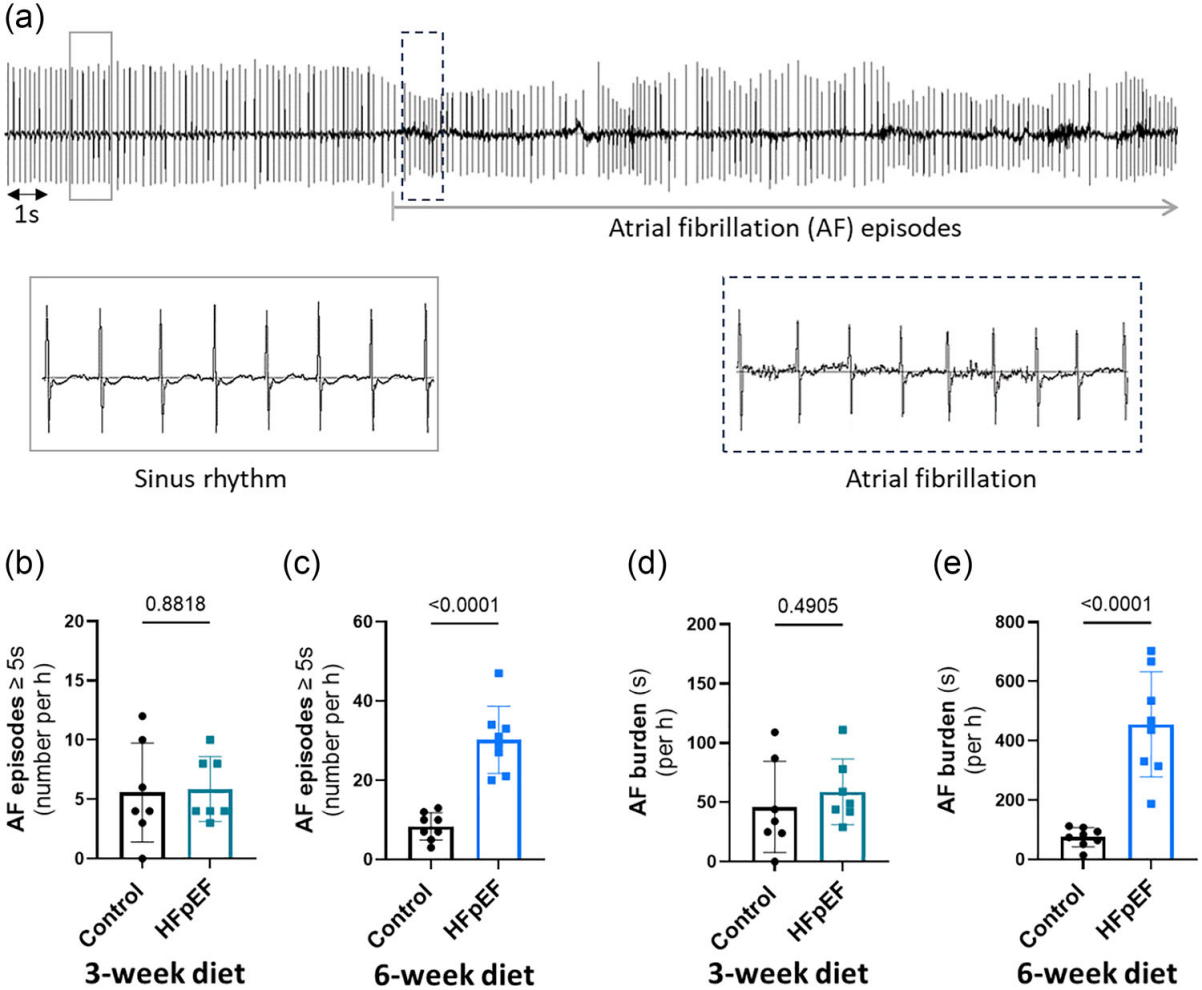
The susceptibility to atrial fibrillation (AF) after isoprenaline (Iso) is increased in the early phase of heart failure with preserved ejection fraction (HFpEF). (a) Representative ECG trace after Iso in HFpEF showing episodes of AF. (b,c) Mean data comparing the number of AF episodes ≥5 s per hour between control and HFpEF groups after 3 weeks (b) and 6 weeks (c) of the diet. (d,e) Mean data comparing the AF burden as the cumulative duration of AF during 1 h between control and HFpEF groups after 3 weeks (d) and 6 weeks (e) of the diet. Experiments were performed in seven (*n* = 7, 3‐week diet) and eight (*n* = 8, 6‐week diet) mice in each group. Values are the mean ± SD. Differences between groups were analysed with Student's two‐tailed *t‐*test. Numbers above bars show *p*‐values.

Next, we investigated whether the susceptibility to atrial arrhythmias, and specifically AF, could be detected at such an early phase of HFpEF. The number of episodes (Figure 4b,c) and the AF burden, characterized by the total duration (Figure 4d,e) of AF during 1 h after Iso injection, was similar after 3 weeks, but significantly higher in the HFpEF group compared with the control group after 6 weeks of the dietary regimen. These data (Figure 4) show that the dietary regimen led to significant susceptibility to AF in the HFpEF group after 6 weeks.

**FIGURE 5:**
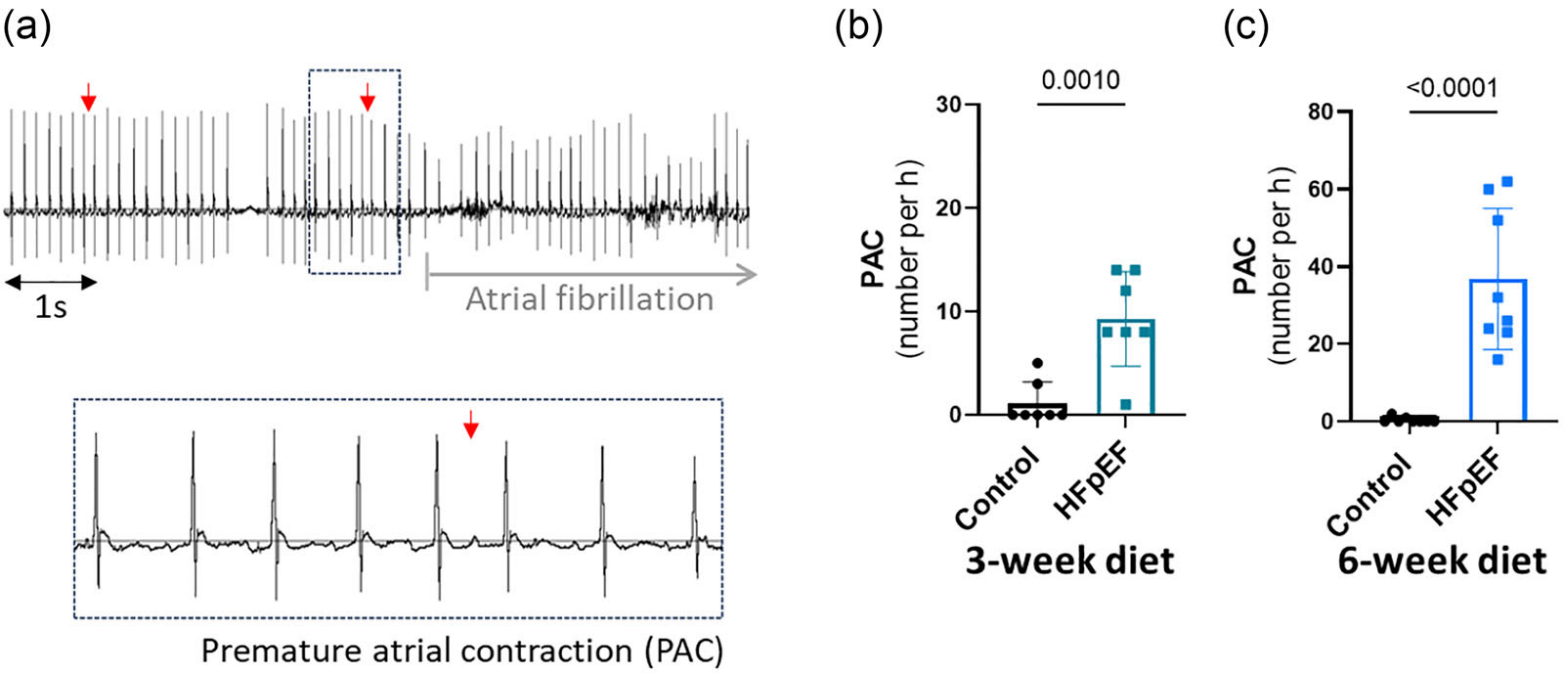
The susceptibility to premature atrial contraction (PAC) after isoprenaline (Iso) is increased in the early phase of heart failure with preserved ejection fraction (HFpEF). (a) Representative ECG trace after Iso in HFpEF showing representative PAC (red arrows) preceding atrial fibrillation (AF) in HFpEF. (b,c) Mean data comparing the number of PAC per hour between control and HFpEF groups after 3 weeks (b) and 6 weeks (c) of the diet. Experiments were performed in seven (*n* = 7, 3‐week diet) and eight (*n* = 8, 6‐week diet) mice in each group. Values are the mean ± SD. Differences between groups were analysed with Student's two‐tailed *t‐*test. Numbers above bars show *p*‐values.

Because premature atrial contractions (PACs) frequently initiate AF (Haissaguerre et al., 1998) and their frequency predicts AF (Dewland et al., 2013), we also searched for PACs in each trace. Figure 4 shows that the number of PACs was significantly higher in the HFpEF group at 3 weeks of the dietary regimen (Figure 5b). The number of PACs was increased further at 6 weeks of the dietary regimen (Figure 5c). Of note, PACs were often present at the initiation of AF episodes after 6 weeks of the dietary regimen. These data suggest that atrial contractile dysfunction that can be characterized by PACs precedes the occurrence of AF in the HFpEF group. Histological evaluation of atria revealed increased atrial size (Figure 3b,c), atrial cardiomyocyte size (Table 1), fibrosis (Figure 3j,k) and apoptosis (Figure 3f,g) in the HFpEF group. These morphological, structural and injury features in atria have been reported to be associated with atrial contractile dysfunction and AF (Nattel et al., 2008; Yamaguchi, 2025). We also evaluated ventricular arrhythmias by searching in the ECG traces for ventricular ectopic beats or ventricular extrasystoles. The HFpEF group of mice did not reveal a significant change in the number of ventricular arrhythmic events compared with the control group at 3 or 6 weeks of the dietary regimen (Table 2).

### 3.3 Susceptibility to conduction system abnormalities and chronotropic incompetence

**FIGURE 6:**
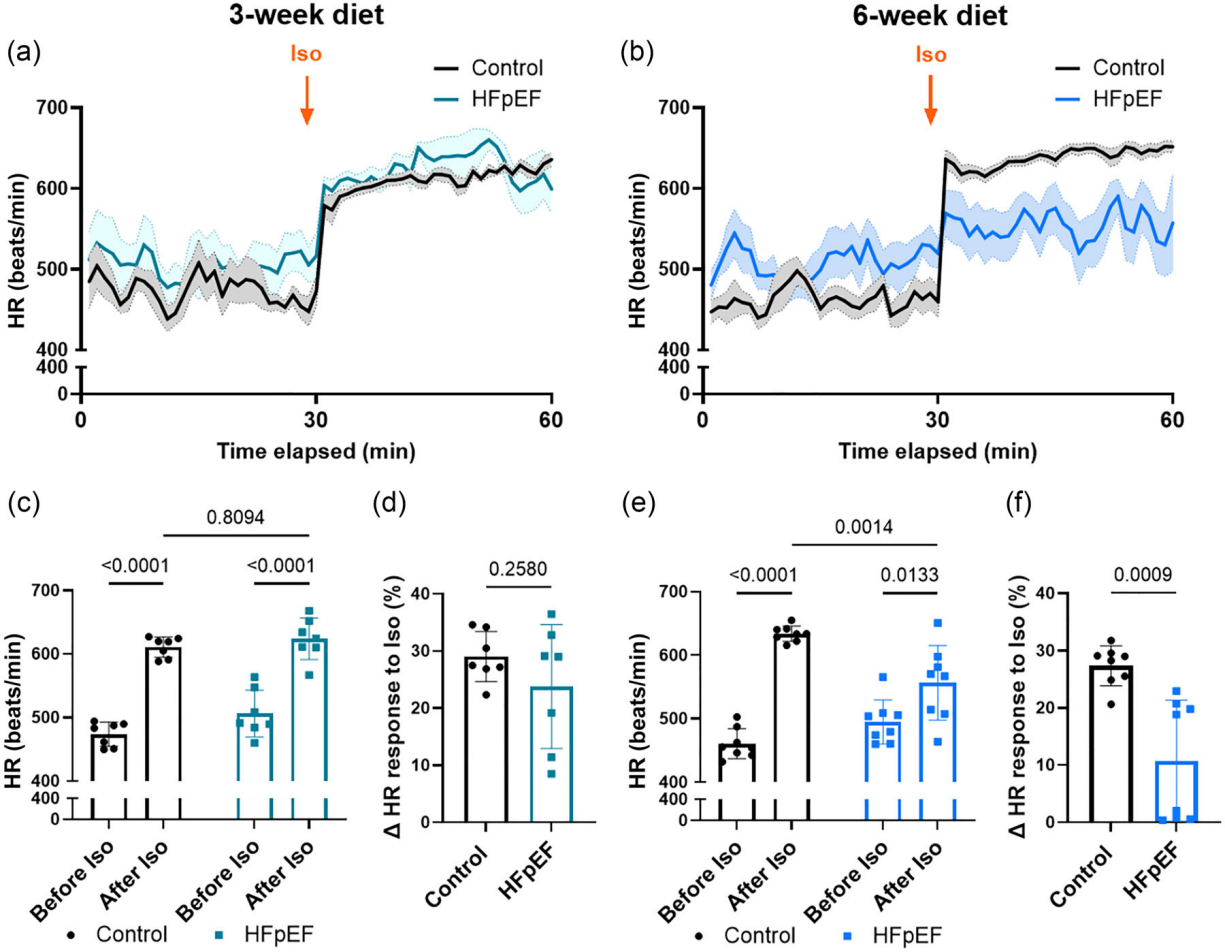
The chronotropic response is impaired in the early phase of heart failure with preserved ejection fraction (HFpEF). (a,b) Curves showing the average evolution of heart rate (HR) from 30 min before isoprenaline (Iso) injection to 30 min after Iso injection, at 3 weeks (a) and 6 weeks (b) after the start of the diet, in control and HFpEF groups. (c–f) Mean data comparing HR before and after Iso (c,e) and the chronotropic response as the percentage HR increase after Iso (d,f) after 3 weeks (c,d) and 6 (weeks e,f) of the dietary regimen. Experiments were performed in seven (*n* = 7, 3‐week diet) and eight (*n* = 8, 6‐week diet) mice in each group. Values are the mean ± SD. Differences among groups were analysed by ordinary two‐way ANOVA followed by the Holm–Sidak *post hoc* test (c,e) or Student's two‐tailed *t‐*test (d,f). Numbers above bars show *p*‐values.

It has been reported that patients with an inadequate heart rate response during exercise (chronotropic incompetence) have an increased risk for developing AF (O'Neal et al., 2016). Therefore, we sought to investigate whether the observed AF in the two‐hit mouse model was associated with chronotropic incompetence. Analysing the ECG trace after Iso injection, we found a comparable significant ability of Iso to increase the HR in both control and HFpEF groups after 3 weeks of the dietary regimen (Figure 6a,c). However, at 6 weeks, the ability of Iso to increase HR in the HFpEF group was significantly reduced (Figure 6b,e). Furthermore, the percentage of HR rise after Iso comparatively to HR recorded before Iso was similar after 3 weeks of the dietary regimen (Figure 6d) and significantly lower, by ∼60%, in the HFpEF group compared with the control group after 6 weeks (Figure 6f). These data show a compromised chronotropic competence simultaneously associated with diastolic dysfunction and AF.

**FIGURE 7:**
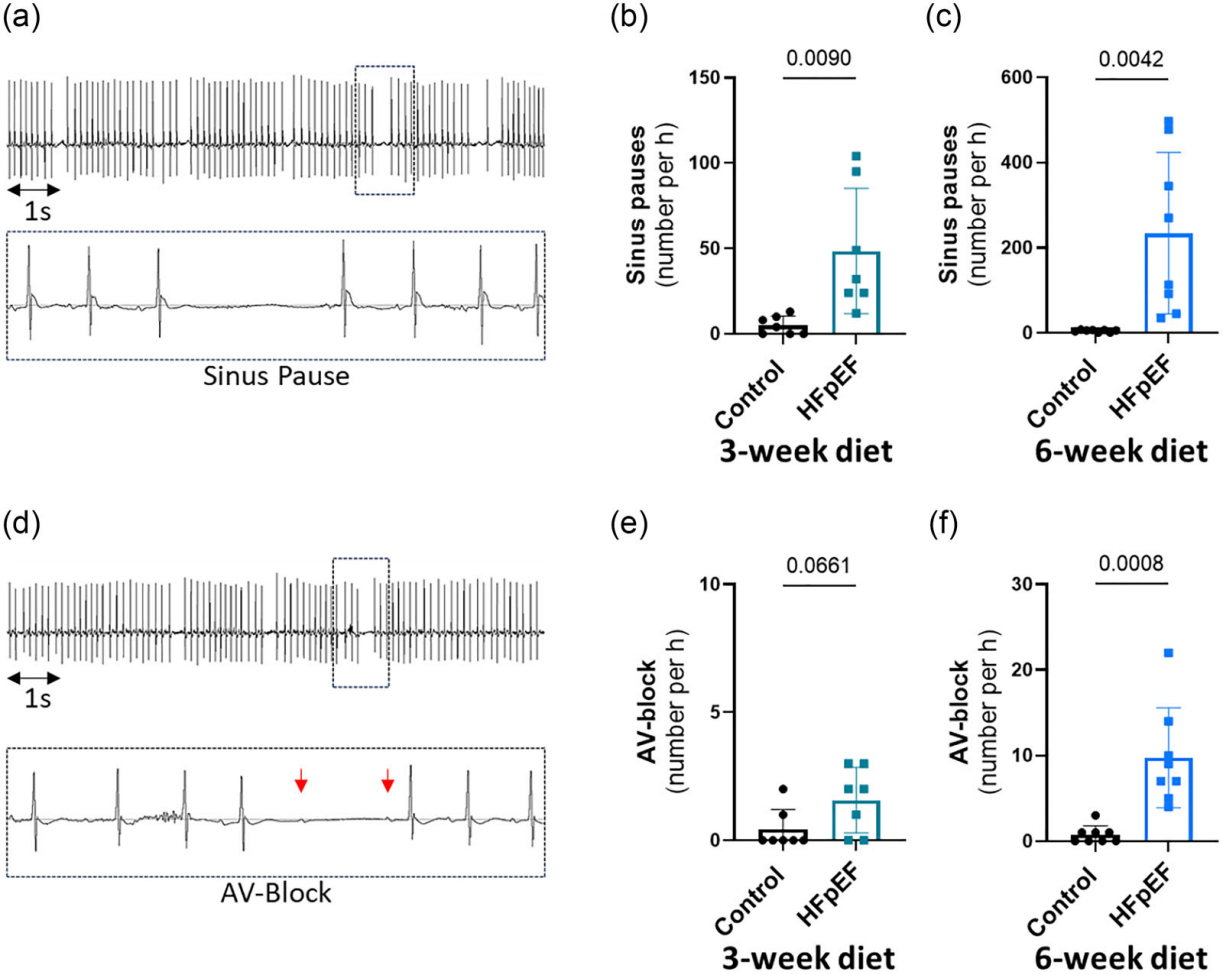
Sinoatrial node (SAN) and atrioventricular node (AVN) signal is impaired after isoprenaline (Iso) in the early phase of heart failure with preserved ejection fraction (HFpEF). (a) Representative ECG trace after Iso in HFpEF showing sinus pauses. (b,c) Mean data comparing the number of sinus pauses per hour after Iso, between control and HFpEF groups after 3 weeks (b) and 6 weeks (c) of dietary regimens. (d) Representative ECG trace after Iso in HFpEF, showing atrioventricular blocks (AV blocks). (e,f) Mean data comparing the number of AV blocks per hour after Iso, between control and HFpEF groups after 3 weeks (e) and 6 weeks (f) of different dietary regimens. Experiments were performed in seven (*n* = 7, 3‐week diet) and eight (*n* = 8, 6‐week diet) mice in each group. Values are the mean ± SD. Differences between groups were analysed with Student's two‐tailed *t‐*test. Numbers above bars show *p*‐values.

Because chronotropic incompetence might be the consequence of reduced SAN function, we investigated the SAN, which regulates the impulse discharge. We found compromised function of the SAN characterized by significantly higher number of sinus pauses, as shown in Figure 7a–c. Interestingly, the number of sinus pauses was already significantly higher at 3 weeks of the dietary regimen (Figure 7b). These data showed early SAN dysfunction, which is likely to be involved in the later chronotropic incompetence and susceptibility to AF. AVN function was impaired in a similar manner to the SAN, as shown by the higher number of AV blocks in Figure 7d–f.

### 3.4 Functioning of sympathetic and parasympathetic limbs of the autonomic nervous system

**FIGURE 8:**
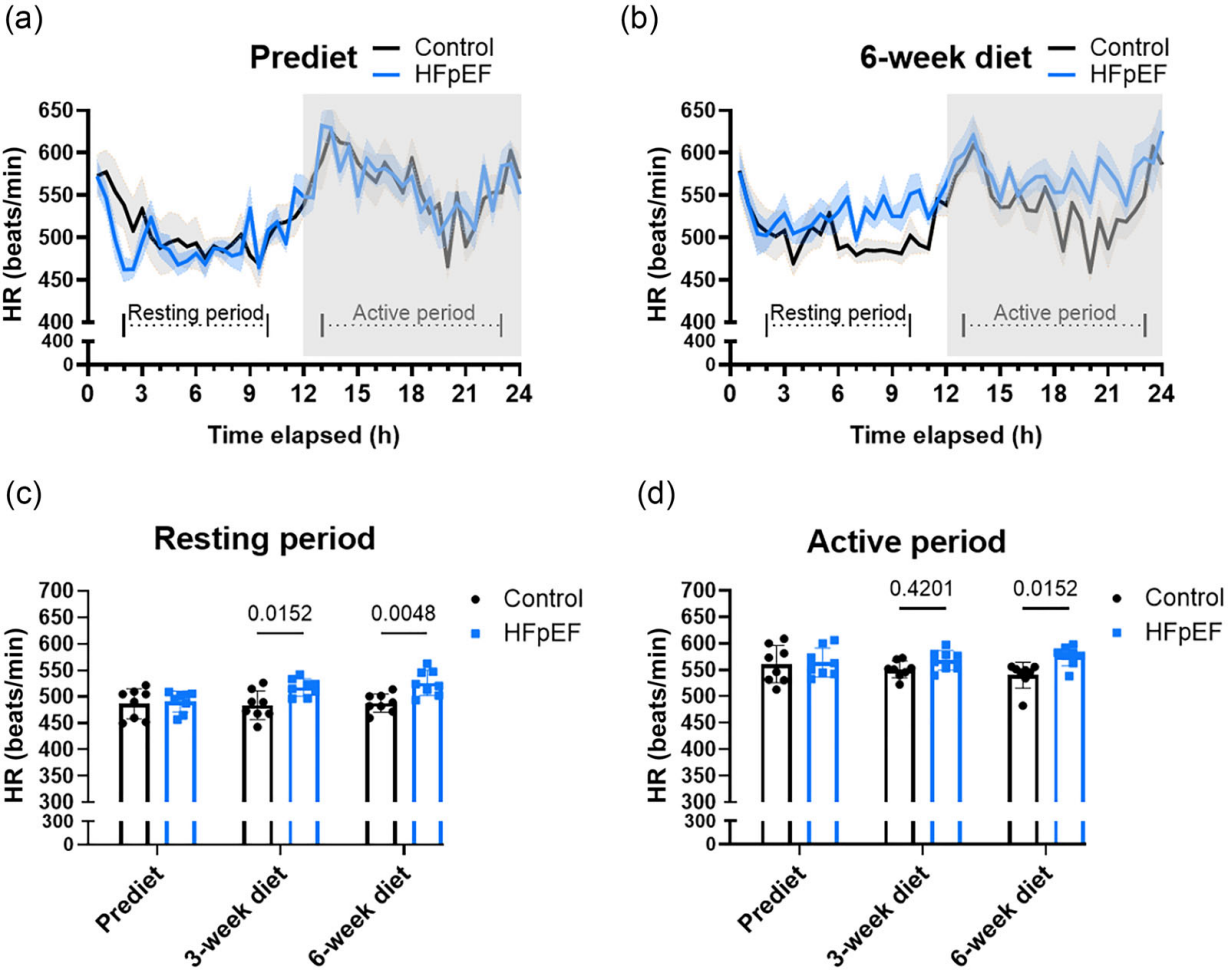
Sympathetic predominance in the early phase of heart failure with preserved ejection fraction (HFpEF). (a,b) The 24 h average trace of heart rate (HR) including resting and active phase before (a) and after 6 weeks (b) of different dietary regimens in control and HFpEF groups. (c,d) Mean data comparing HR between control and HFpEF groups during resting (c) and active (d) phases before and at the indicated recording times after initiation of the dietary regimen. Experiments were performed in eight (*n* = 8) mice in each group. Values are the mean ± SD. Differences between groups were analysed by ordinary two‐way ANOVA followed by the Holm–Sidak *post hoc* test. Numbers above bars show *p*‐values.

We also investigated events preceding AF and diastolic function by exploring the elevated resting sympathetic activity that is commonly seen in patients with HFpEF and can contribute to diastolic dysfunction (Ariyaratnam et al., 2021; Piccirillo et al., 2006; Somsen et al., 1996). Autonomic nervous system dysfunction associated with elevated sympathetic activity is also reported to play a pivotal role in initiating and maintaining cardiac arrhythmia, including AF (Chen et al., 2014; Shen & Zipes, 2014). Initially, we explored the dynamic balance between the sympathetic and parasympathetic divisions of the autonomic nervous system for 24 h, including active and resting phases. From 3 weeks of the dietary regimen, the heart rate during the resting phase was significantly higher in the HFpEF compared with the control group (Figure 8c). In the active phase, however, the elevated HR appeared to be significant only after 6 weeks of the dietary regimen (Figure 8d). The higher HR was corroborated by the lower RR interval in the same mice (Table 2). As shown in Table 2, SDNN, which reflects the overall HR variability, and root mean square of successive diffrences between normal heartbeats, which reflects the vagally mediated changes indicated in HR variability, were significantly lower after the dietary regimen, suggesting lower HR variability and lower parasympathetic activity, hence a likely enhanced sympathetic drive or activity that is shown with increased HR (Figure 8). Taken together, sympathetic predominance appears early in the mouse HFpEF of our study.

## 4 DISCUSSION

In this study, we have characterized, in a time‐dependent manner, several clinically known features of HFpEF associated with AF in a mouse HFpEF model. We showed that diastolic dysfunction, chronotropic incompetence and susceptibility to AF occur simultaneously in the early phase of HFpEF (6 weeks of HFD + l‐NAME). We also showed that these key features of HFpEF were preceded (3 weeks of HFD + l‐NAME) by autonomic imbalance and susceptibility to premature atrial contraction and conduction abnormalities.

Initially, we verified the capacity of HFD and l‐NAME to induce the two common risk factors of HFpEF and found, as expected, a progressive significant increase of body weight and blood pressure after initiation of the dietary regimen (Figure 1). However, the lung weight (Table 1), indicating congestion, considered as a primary feature to confirm heart failure (Roh et al., 2022), was only slightly, although significantly, increased compared with the control group. The locomotive activity (Table 2), which can reflect signs of exhaustion, was comparable between control and HFpEF groups. Nevertheless, the diastolic dysfunction, which is regarded as a central characteristic of HFpEF (Zile et al., 2004), and LV hypertrophy were significantly more pronounced in the HFpEF group after 6 weeks of the dietary regimen, as shown in Figures 2 and 3 and Table 1. Although diastolic dysfunction was found at 6 weeks of the dietary regimen, this phenotype was not present at 3 weeks, confirming its progressive nature. Because diastolic dysfunction usually progresses to overt HFpEF, we concluded that 6 weeks after the initiation of the dietary regimen in our model corresponds to an early phase of HFpEF. In a similar manner to diastolic dysfunction, the susceptibility to AF was absent at 3 weeks but significantly increased at 6 weeks of the dietary regimen (Figure 4). These data are consistent with clinical reports that the prevalence and progression of diastolic dysfunction are higher in patients with AF (Naser et al., 2023; Reddy et al., 2018). Additionally, patients without a diagnosis of HFpEF who present with AF are reported commonly to have increased LV filling pressures or diastolic dysfunction (Reddy et al., 2018), a central characteristic of HFpEF (Zile et al., 2004). Therefore, the model in the present study might be considered for studying the early stages of AF and diastolic dysfunction. These observations also reveal a simultaneous and somewhat parallel progression of AF and diastolic dysfunction in the model and therefore point towards the possible contribution of the same underlying driver for the two disorders. This is in line with the third of the suggested pathophysiological explanations derived from epidemiological and clinical studies on interdependence between AF and HFpEF, as presented in the Introduction (Packer et al., 2020). However, the other possible explanations based on mutual causality between AF and diastolic dysfunction cannot be ruled out. Our data showing increased LA size support the possible impact of LV stiffness on atrial remodelling and, subsequently, AF. However, increased RA size and remodelling, in addition to increased cell death in both LA and RA, was also observed (Figure 3), suggesting broader changes in the atria, potentially with different causes.

Strong evidence for the involvement of PACs in the occurrence of AF has been established in patients with paroxysmal AF, both with non‐prevalent and prevalent AF attacks (Dewland et al., 2013; Haissaguerre et al., 1998). In our study, higher susceptibility to PACs was observed, and PACs were often found before the initiation of AF on the ECG trace (Figure 5). These observations suggest that atrial contractile dysfunction might be the result of atrial remodelling, potentially subsequent to diastolic stiffness, and could therefore be the link between diastolic stiffness and AF. However, susceptibility to PACs was already significantly higher at 3 weeks of the dietary regimen, when diastolic dysfunction and susceptibility to AF were not changed. Additionally, remodelling and cell death in both RA and LA have been reported to lead to atrial dysfunction and AF (Aime‐Sempe et al., 1999; Jalife & Kaur, 2015; Nattel & Harada, 2014). These results suggest a different cause or at least another contributor to PACs. A study using isolated rabbit atrial sections has suggested electrical modulation interaction between SAN tissue, atrial myocardium and pulmonary vein tissue (often the origin of AF) (Chen et al., 2014). SAN dysfunction is also reported to increase the occurrence of AF (Dobrzynski et al., 2007; John & Kumar, 2016). Of interest, we found that other conduction abnormalities, such as an increased number of sinus pauses (SAN dysfunction) and AV block (Figure 7), were present at 3 weeks of the dietary regimen, similar to PACs. Therefore, it is conceivable that a stressor other than ventricular stiffness contributes to SAN dysfunction and PACs that might subsequently promote AF. The occurrence of conduction abnormalities is in line with a study by Mesquita et al. (2022) reporting conduction blocks in isolated SAN preparations from the same mouse model of HFpEF (Mesquita et al., 2022). In the study by Mesquita et al. (2022), SAN dysfunction was associated with impaired SAN β‐adrenergic receptor responsiveness and uncoordinated recruitment of pacemaker clusters favouring rhythm abnormalities (Mesquita et al., 2022). At 6 weeks of the dietary regimen, we found significant chronotropic incompetence after β‐adrenergic receptor challenge with Iso (Figure 6). Epidemiological reports suggest that patients with AF and without diagnosis of HFpEF commonly have increased filling pressure, and in patients with exertional dyspnoea, the presence of AF was a predictor of underlying HFpEF (Reddy et al., 2018). Additionally, an SAN defect attributable to impaired SAN β‐receptor function has been suggested to be responsible, at least in part, for the chronotropic incompetence commonly seen in HF patients (Sarma et al., 2020). Concomitantly, Mesquita et al. (2022) reported an attenuated beating rate in response to Iso in the SAN preparation (Mesquita et al., 2022). Our findings of parallel occurrence of PAC, SAN dysfunction and AF with diastolic dysfunction in this mouse model are consistent with the clinically recognized association of AF and HFpEF.

One of the most common features of HFpEF that has been linked with the clinical characteristics above and revealed in the present mouse model of HFpEF is autonomic imbalance characterized by sympathetic predominance. Accordingly, we found in our recordings a significant resting sympathetic predominance in the model after 3 weeks of the dietary regimen. We also found that the increased resting sympathetic activity, although not significant, was already trending to be higher after 1 week of the dietary regimen (data not shown). The sympathetic predominance and autonomic imbalance resulting from vagal withdrawal is a common feature of human HFpEF (Castiglione et al., 2023; Floras & Ponikowski, 2015). The autonomic imbalance has been shown to contribute to an impaired chronotropic response (Borlaug et al., 2006). Several animal and human studies have demonstrated that the autonomic nervous system plays a central role in the initiation and maintenance of AF, especially in the early stages, when activation of the extrinsic autonomic nervous system of the heart or intrinsic cardiac nerve activity is seen prior to AF and tachyarrhythmia onset in 73%–100% of cases (Choi et al., 2010; Tan et al., 2008). Additionally, several preclinical and clinical studies have shown a relationship between an elevated sympathetic activity and the development of diastolic dysfunction (Ariyaratnam et al., 2021; Piccirillo et al., 2006; Somsen et al., 1996). Hence, the presence of autonomic imbalance further supports the capability of this mouse model of HFpEF to recapitulate key features of clinical HFpEF associated with AF. Additionally, the early occurrence of sympathetic predominance in the model suggests its potential role in driving or mediating in parallel both AF and diastolic dysfunction.

## 5 CONCLUSION

We uncovered evidence showing some similarity between the development of AF in the mouse model of HFpEF and clinical HFpEF associated with AF. In this mouse model, we characterized a time‐dependent development of AF and diastolic dysfunction, in addition to important features of clinical HFpEF linked to the two disorders. Although it was not the scope of our study to establish the causal relationship between the identified features, our data might form the basis for addressing this limitation in future studies. Our study indicates that this model might be helpful for studies addressing the mechanism, burden, heterogeneity and multidimensional nature of the interconvergence between AF and HFpEF. The present study might also be viewed as a basis for using the present mouse model for studying the early manifestation and prevention of HFpEF associated with AF.

## AUTHOR CONTRIBUTIONS

Bernadin Ndongson‐Dongmo and Finn Olav Levy designed research; Bernadin Ndongson‐Dongmo performed research; Bernadin Ndongson‐Dongmo, Reinhard Bauer and Finn Olav Levy analysed data; Bernadin Ndongson‐Dongmo wrote the manuscript. All authors revised and approved the final version of the manuscript and agree to be accountable for all aspects of the work in ensuring that questions related to the accuracy or integrity of any part of the work are appropriately investigated and resolved. All persons designated as authors qualify for authorship, and all those who qualify for authorship are listed.

## CONFLICT OF INTEREST

None declared.

## Data Availability

All data supporting the results are in the publication. All original data in this publication are available upon reasonable request to the corresponding authors.

## References

[eph70019-bib-0001] Aime‐Sempe, C. , Folliguet, T. , Rucker‐Martin, C. , Krajewska, M. , Krajewska, S. , Heimburger, M. , Aubier, M. , Mercadier, J. J. , Reed, J. C. , & Hatem, S. N. (1999). Myocardial cell death in fibrillating and dilated human right atria. Journal of the American College of Cardiology, 34(5), 1577–1586. 10551709 10.1016/s0735-1097(99)00382-4

[eph70019-bib-0002] Ariyaratnam, J. P. , Elliott, A. D. , Mishima, R. S. , Gallagher, C. , Lau, D. H. , & Sanders, P. (2021). Heart failure with preserved ejection fraction: An alternative paradigm to explain the clinical implications of atrial fibrillation. Heart Rhythm O2, 2(6), 771–783. 34988529 10.1016/j.hroo.2021.09.015 PMC8710629

[eph70019-bib-0003] Bapat, A. , Schloss, M. J. , Yamazoe, M. , Grune, J. , Hulsmans, M. , Milan, D. J. , Nahrendorf, M. , & Ellinor, P. T. (2023). A mouse model of atrial fibrillation in sepsis. Circulation, 147(13), 1047–1049. 36972346 10.1161/CIRCULATIONAHA.122.060317 PMC10057612

[eph70019-bib-0004] Borlaug, B. A. , Melenovsky, V. , Russell, S. D. , Kessler, K. , Pacak, K. , Becker, L. C. , & Kass, D. A. (2006). Impaired chronotropic and vasodilator reserves limit exercise capacity in patients with heart failure and a preserved ejection fraction. Circulation, 114(20), 2138–2147. 17088459 10.1161/CIRCULATIONAHA.106.632745

[eph70019-bib-0005] Castiglione, V. , Gentile, F. , Ghionzoli, N. , Chiriaco, M. , Panichella, G. , Aimo, A. , Vergaro, G. , Giannoni, A. , Passino, C. , & Emdin, M. (2023). Pathophysiological rationale and clinical evidence for neurohormonal modulation in heart failure with preserved ejection fraction. Cardiac Failure Review, 9, e09. 37427009 10.15420/cfr.2022.23 PMC10326668

[eph70019-bib-0006] Chen, P. S. , Chen, L. S. , Fishbein, M. C. , Lin, S. F. , & Nattel, S. (2014). Role of the autonomic nervous system in atrial fibrillation: Pathophysiology and therapy. Circulation Research, 114(9), 1500–1515. 24763467 10.1161/CIRCRESAHA.114.303772 PMC4043633

[eph70019-bib-0007] Chen, Y. C. , Lu, Y. Y. , Cheng, C. C. , Lin, Y. K. , Chen, S. A. , & Chen, Y. J. (2014). Sinoatrial node electrical activity modulates pulmonary vein arrhythmogenesis. International Journal of Cardiology, 173(3), 447–452. 24681021 10.1016/j.ijcard.2014.03.009

[eph70019-bib-0008] Choi, E. K. , Shen, M. J. , Han, S. , Kim, D. , Hwang, S. , Sayfo, S. , Piccirillo, G. , Frick, K. , Fishbein, M. C. , Hwang, C. , Lin, S. F. , & Chen, P. S. (2010). Intrinsic cardiac nerve activity and paroxysmal atrial tachyarrhythmia in ambulatory dogs. Circulation, 121(24), 2615–2623. 20529998 10.1161/CIRCULATIONAHA.109.919829 PMC2890034

[eph70019-bib-0009] Dewland, T. A. , Vittinghoff, E. , Mandyam, M. C. , Heckbert, S. R. , Siscovick, D. S. , Stein, P. K. , Psaty, B. M. , Sotoodehnia, N. , Gottdiener, J. S. , & Marcus, G. M. (2013). Atrial ectopy as a predictor of incident atrial fibrillation: A cohort study. Annals of Internal Medicine, 159(11), 721–728. 24297188 10.7326/0003-4819-159-11-201312030-00004 PMC4115459

[eph70019-bib-0010] Dobrzynski, H. , Boyett, M. R. , & Anderson, R. H. (2007). New insights into pacemaker activity: Promoting understanding of sick sinus syndrome. Circulation, 115(14), 1921–1932. 17420362 10.1161/CIRCULATIONAHA.106.616011

[eph70019-bib-0011] Doi, R. , Masuyama, T. , Yamamoto, K. , Doi, Y. , Mano, T. , Sakata, Y. , Ono, K. , Kuzuya, T. , Hirota, S. , Koyama, T. , Miwa, T. , & Hori, M. (2000). Development of different phenotypes of hypertensive heart failure: Systolic versus diastolic failure in Dahl salt‐sensitive rats. Journal of Hypertension, 18(1), 111–120. 10678551 10.1097/00004872-200018010-00016

[eph70019-bib-0012] Floras, J. S. , & Ponikowski, P. (2015). The sympathetic/parasympathetic imbalance in heart failure with reduced ejection fraction. European Heart Journal, 36(30), 1974–1982b. 25975657 10.1093/eurheartj/ehv087 PMC4528097

[eph70019-bib-0013] Gentile, F. , Ghionzoli, N. , Borrelli, C. , Vergaro, G. , Pastore, M. C. , Cameli, M. , Emdin, M. , Passino, C. , & Giannoni, A. (2022). Epidemiological and clinical boundaries of heart failure with preserved ejection fraction. European Journal of Preventive Cardiology, 29(8), 1233–1243. 33963839 10.1093/eurjpc/zwab077

[eph70019-bib-0014] Haissaguerre, M. , Jais, P. , Shah, D. C. , Takahashi, A. , Hocini, M. , Quiniou, G. , Garrigue, S. , Le Mouroux, A. , Le Metayer, P. , & Clementy, J. (1998). Spontaneous initiation of atrial fibrillation by ectopic beats originating in the pulmonary veins. New England Journal of Medicine, 339(10), 659–666. 9725923 10.1056/NEJM199809033391003

[eph70019-bib-0015] Hamdani, N. , Franssen, C. , Lourenco, A. , Falcao‐Pires, I. , Fontoura, D. , Leite, S. , Plettig, L. , Lopez, B. , Ottenheijm, C. A. , Becher, P. M. , Gonzalez, A. , Tschope, C. , Diez, J. , Linke, W. A. , Leite‐Moreira, A. F. , & Paulus, W. J. (2013). Myocardial titin hypophosphorylation importantly contributes to heart failure with preserved ejection fraction in a rat metabolic risk model. Circulation Heart Failure, 6(6), 1239–1249. 24014826 10.1161/CIRCHEARTFAILURE.113.000539

[eph70019-bib-0016] Heijman, J. , Luermans, J. , Linz, D. , van Gelder, I. C. , & Crijns, H. (2021). Risk factors for atrial fibrillation progression. Cardiac Electrophysiology Clinics, 13(1), 201–209. 33516398 10.1016/j.ccep.2020.10.011

[eph70019-bib-0017] Hoit, B. D. (2014). Left atrial size and function: Role in prognosis. Journal of the American College of Cardiology, 63(6), 493–505. 24291276 10.1016/j.jacc.2013.10.055

[eph70019-bib-0018] Jalife, J. , & Kaur, K. (2015). Atrial remodeling, fibrosis, and atrial fibrillation. Trends in Cardiovascular Medicine, 25(6), 475–484. 25661032 10.1016/j.tcm.2014.12.015 PMC5658790

[eph70019-bib-0019] John, R. M. , & Kumar, S. (2016). Sinus node and atrial arrhythmias. Circulation, 133(19), 1892–1900. 27166347 10.1161/CIRCULATIONAHA.116.018011

[eph70019-bib-0020] Kondo, H. , Abe, I. , Gotoh, K. , Fukui, A. , Takanari, H. , Ishii, Y. , Ikebe, Y. , Kira, S. , Oniki, T. , Saito, S. , Aoki, K. , Tanino, T. , Mitarai, K. , Kawano, K. , Miyoshi, M. , Fujinami, M. , Yoshimura, S. , Ayabe, R. , Okada, N. , … & Takahashi, N. (2018). Interleukin 10 treatment ameliorates high‐fat diet‐induced inflammatory atrial remodeling and fibrillation. Circulation: Arrhythmia and Electrophysiology, 11(5), e006040. 29748196 10.1161/CIRCEP.117.006040

[eph70019-bib-0021] Kotecha, D. , Lam, C. S. , Van Veldhuisen, D. J. , Van Gelder, I. C. , Voors, A. A. , & Rienstra, M. (2016). Heart failure with preserved ejection fraction and atrial fibrillation: Vicious twins. Journal of the American College of Cardiology, 68(20), 2217–2228. 27855811 10.1016/j.jacc.2016.08.048

[eph70019-bib-0022] Mesquita, T. , Zhang, R. , Cho, J. H. , Zhang, R. , Lin, Y. N. , Sanchez, L. , Goldhaber, J. I. , Yu, J. K. , Liang, J. A. , Liu, W. , Trayanova, N. A. , & Cingolani, E. (2022). Mechanisms of sinoatrial node dysfunction in heart failure with preserved ejection fraction. Circulation, 145(1), 45–60. 34905696 10.1161/CIRCULATIONAHA.121.054976 PMC9083886

[eph70019-bib-0023] Naser, J. A. , Lee, E. , Scott, C. G. , Kennedy, A. M. , Pellikka, P. A. , Lin, G. , Pislaru, S. V. , & Borlaug, B. A. (2023). Prevalence and incidence of diastolic dysfunction in atrial fibrillation: Clinical implications. European Heart Journal, 44(48), 5049–5060. 37639219 10.1093/eurheartj/ehad592 PMC13386646

[eph70019-bib-0024] Nattel, S. , Burstein, B. , & Dobrev, D. (2008). Atrial remodeling and atrial fibrillation: Mechanisms and implications. Circulation: Arrhythmia and Electrophysiology, 1(1), 62–73. 19808395 10.1161/CIRCEP.107.754564

[eph70019-bib-0025] Nattel, S. , & Harada, M. (2014). Atrial remodeling and atrial fibrillation: Recent advances and translational perspectives. Journal of the American College of Cardiology, 63(22), 2335–2345. 24613319 10.1016/j.jacc.2014.02.555

[eph70019-bib-0026] O'Neal, W. T. , Qureshi, W. T. , Blaha, M. J. , Dardari, Z. A. , Ehrman, J. K. , Brawner, C. A. , Soliman, E. Z. , & Al‐Mallah, M. H. (2016). Chronotropic incompetence and risk of atrial fibrillation: The Henry Ford exercise testing (FIT) Project. JACC Clinical Electrophysiology, 2(6), 645–652. 28451646 10.1016/j.jacep.2016.03.013 PMC5403158

[eph70019-bib-0027] Pacher, P. , Nagayama, T. , Mukhopadhyay, P. , Batkai, S. , & Kass, D. A. (2008). Measurement of cardiac function using pressure‐volume conductance catheter technique in mice and rats. Nature Protocols, 3(9), 1422–1434. 18772869 10.1038/nprot.2008.138 PMC2597499

[eph70019-bib-0028] Packer, M. , Lam, C. S. P. , Lund, L. H. , & Redfield, M. M. (2020). Interdependence of atrial fibrillation and heart failure with a preserved ejection fraction reflects a common underlying atrial and ventricular myopathy. Circulation, 141(1), 4–6. 31887078 10.1161/CIRCULATIONAHA.119.042996

[eph70019-bib-0029] Paulus, W. J. , & Tschope, C. (2013). A novel paradigm for heart failure with preserved ejection fraction: Comorbidities drive myocardial dysfunction and remodeling through coronary microvascular endothelial inflammation. Journal of the American College of Cardiology, 62(4), 263–271. 23684677 10.1016/j.jacc.2013.02.092

[eph70019-bib-0030] Pfeffer, M. A. , Shah, A. M. , & Borlaug, B. A. (2019). Heart failure with preserved ejection fraction in perspective. Circulation Research, 124(11), 1598–1617. 31120821 10.1161/CIRCRESAHA.119.313572 PMC6534165

[eph70019-bib-0031] Piccirillo, G. , Germano, G. , Vitarelli, A. , Ragazzo, M. , di Carlo, S. , De Laurentis, T. , Torrini, A. , Matera, S. , Magnanti, M. , Marchitto, N. , Bonanni, L. , & Magri, D. (2006). Autonomic cardiovascular control and diastolic dysfunction in hypertensive subjects. International Journal of Cardiology, 110(2), 160–166. 16051387 10.1016/j.ijcard.2005.06.041

[eph70019-bib-0032] Polina, I. , Jansen, H. J. , Li, T. , Moghtadaei, M. , Bohne, L. J. , Liu, Y. , Krishnaswamy, P. , Egom, E. E. , Belke, D. D. , Rafferty, S. A. , Ezeani, M. , Gillis, A. M. , & Rose, R. A. (2020). Loss of insulin signaling may contribute to atrial fibrillation and atrial electrical remodeling in type 1 diabetes. PNAS, 117(14), 7990–8000. 32198206 10.1073/pnas.1914853117 PMC7148583

[eph70019-bib-0033] Reddy, Y. N. V. , Obokata, M. , Gersh, B. J. , & Borlaug, B. A. (2018). High prevalence of occult heart failure with preserved ejection fraction among patients with atrial fibrillation and dyspnea. Circulation, 137(5), 534–535. 29378762 10.1161/CIRCULATIONAHA.117.030093 PMC5793884

[eph70019-bib-0034] Riley, G. , Syeda, F. , Kirchhof, P. , & Fabritz, L. (2012). An introduction to murine models of atrial fibrillation. Frontiers in Physiology, 3, 296. 22934047 10.3389/fphys.2012.00296 PMC3429067

[eph70019-bib-0035] Roh, J. , Hill, J. A. , Singh, A. , Valero‐Munoz, M. , & Sam, F. (2022). Heart failure with preserved ejection fraction: Heterogeneous syndrome, diverse preclinical models. Circulation Research, 130(12), 1906–1925. 35679364 10.1161/CIRCRESAHA.122.320257 PMC10035274

[eph70019-bib-0036] Sarma, S. , Stoller, D. , Hendrix, J. , Howden, E. , Lawley, J. , Livingston, S. , Adams‐Huet, B. , Holmes, C. , Goldstein, D. S. , & Levine, B. D. (2020). Mechanisms of chronotropic incompetence in heart failure with preserved ejection fraction. Circulation: Heart Failure, 13(3), e006331. 32164435 10.1161/CIRCHEARTFAILURE.119.006331 PMC7347285

[eph70019-bib-0037] Sartipy, U. , Dahlstrom, U. , Fu, M. , & Lund, L. H. (2017). Atrial fibrillation in heart failure with preserved, mid‐range, and reduced ejection fraction. JACC Heart Failure, 5(8), 565–574. 28711451 10.1016/j.jchf.2017.05.001

[eph70019-bib-0038] Schiattarella, G. G. , Altamirano, F. , Tong, D. , French, K. M. , Villalobos, E. , Kim, S. Y. , Luo, X. , Jiang, N. , May, H. I. , Wang, Z. V. , Hill, T. M. , Mammen, P. P. A. , Huang, J. , Lee, D. I. , Hahn, V. S. , Sharma, K. , Kass, D. A. , Lavandero, S. , Gillette, T. G. , & Hill, J. A. (2019). Nitrosative stress drives heart failure with preserved ejection fraction. Nature, 568(7752), 351–356. 30971818 10.1038/s41586-019-1100-z PMC6635957

[eph70019-bib-0039] Shah, S. J. , Borlaug, B. A. , Kitzman, D. W. , McCulloch, A. D. , Blaxall, B. C. , Agarwal, R. , Chirinos, J. A. , Collins, S. , Deo, R. C. , Gladwin, M. T. , Granzier, H. , Hummel, S. L. , Kass, D. A. , Redfield, M. M. , Sam, F. , Wang, T. J. , Desvigne‐Nickens, P. , & Adhikari, B. B. (2020). Research priorities for heart failure with preserved ejection fraction: National heart, lung, and blood institute working group summary. Circulation, 141(12), 1001–1026. 32202936 10.1161/CIRCULATIONAHA.119.041886 PMC7101072

[eph70019-bib-0040] Shen, M. J. , & Zipes, D. P. (2014). Role of the autonomic nervous system in modulating cardiac arrhythmias. Circulation Research, 114(6), 1004–1021. 24625726 10.1161/CIRCRESAHA.113.302549

[eph70019-bib-0041] Somsen, G. A. , Dubois, E. A. , Brandsma, K. , de Jong, J. , van der Wouw, P. A. , Batink, H. D. , van Royen, E. A. , Lie, K. I. , & van Zwieten, P. A. (1996). Cardiac sympathetic neuronal function in left ventricular volume and pressure overload. Cardiovascular Research, 31(1), 132–138. 8849597

[eph70019-bib-0042] Tan, A. Y. , Zhou, S. , Ogawa, M. , Song, J. , Chu, M. , Li, H. , Fishbein, M. C. , Lin, S. F. , Chen, L. S. , & Chen, P. S. (2008). Neural mechanisms of paroxysmal atrial fibrillation and paroxysmal atrial tachycardia in ambulatory canines. Circulation, 118(9), 916–925. 18697820 10.1161/CIRCULATIONAHA.108.776203 PMC2742977

[eph70019-bib-0043] Yamaguchi, T. (2025). Atrial structural remodeling and atrial fibrillation substrate: A histopathological perspective. Journal of Cardiology, 85(2), 47–55. 38810728 10.1016/j.jjcc.2024.05.007

[eph70019-bib-0044] Zile, M. R. , Baicu, C. F. , & Gaasch, W. H. (2004). Diastolic heart failure – Abnormalities in active relaxation and passive stiffness of the left ventricle. New England Journal of Medicine, 350(19), 1953–1959. 15128895 10.1056/NEJMoa032566

